# Anti-ribosomal P (anti-P) antibodies in patients with autoimmune hepatitis

**DOI:** 10.31744/einstein_journal/2023AO0375

**Published:** 2023-11-10

**Authors:** Clarisse de Almeida Gallo, Alessandra Dellavance, Raimundo Araújo Gama, Antônio Eduardo Silva, Ivonete Sandra de Souza e Silva, Luis Eduardo Coelho Andrade, Maria Lúcia Gomez Ferraz

**Affiliations:** 1 Division of Gastroenterology Universidade Federal de São Paulo São Paulo SP Brazil Division of Gastroenterology , Universidade Federal de São Paulo , São Paulo , SP , Brazil .; 2 Research and Development Division Grupo Fleury São Paulo SP Brazil Research and Development Division , Grupo Fleury , São Paulo , SP , Brazil .; 3 Division of Rheumatology Universidade Federal de São Paulo São Paulo SP Brazil Division of Rheumatology , Universidade Federal de São Paulo , São Paulo , SP , Brazil .; 4 Division of Immunology Grupo Fleury São Paulo SP Brazil Division of Immunology , Grupo Fleury , São Paulo , SP , Brazil .

**Keywords:** Hepatitis, autoimmune, Ribosomal proteins, Autoantibodies, Lupus erythematosus, systemic, Enzyme-linked immunosorbent assay, Luminescence

## Abstract

**Objective:**

Published studies have shown associations between anti-ribosomal P (anti-P) antibody and systemic lupus erythematosus with hepatic manifestations. This has been reported also in autoimmune hepatitis. However, the consistency of the latter association remains controversial. This study aimed to evaluate the frequency of anti-P antibodies in autoimmune hepatitis using two different immunoassays.

**Methods:**

One-hundred and seventy-seven patients with autoimmune hepatitis were screened, and 142 were analyzed for anti-P antibody positivity. The samples were first analyzed using two different immunoassays: enzyme-linked immunosorbent assay (ELISA) and chemiluminescence and then compared with a group of 60 patients with systemic lupus erythematous. The positive samples were subjected to western blot analysis.

**Results:**

Anti-P was found in 5/142 autoimmune hepatitis cases (3.5%) by chemiluminescence and in none by ELISA. Among the five chemiluminescence-positive autoimmune hepatitis samples, on anti-P western blot analysis one was negative, two were weakly positive, and two were positive. In contrast, anti-P was detected in 10/60 patients with systemic lupus erythematosus (16.7%) and presented higher chemiluminescence units than the autoimmune hepatitis samples.

**Conclusion:**

A low frequency of anti-P antibodies was observed in autoimmune hepatitis, suggesting that this test is not useful for the diagnosis or management of this disease.

## In Brief

Anti-P-ribosomal antibody is a biomarker of systemic lupus erythematosus mainly associated with renal, nervous, and hepatic involvement. Systemic lupus erythematosus may present with features similar to autoimmune hepatitis. This study aimed to investigate the association of Anti-P-ribosomal antibodies in systemic lupus erythematosus compared to autoimmune hepatitis in the general Brazilian population.

## Highlights۪

Autoimmune hepatitis and systemic lupus erythematosus share several clinical features.

۪Anti-P ribosomal antibody is a biomarker for systemic lupus erythematosus.

The association between anti-P ribosomal antibody and autoimmune hepatitis has shown conflicting results.

Our results showed no association between anti-P ribosomal antibody and autoimmune hepatitis.

## Visual Abstract

**Figure:**
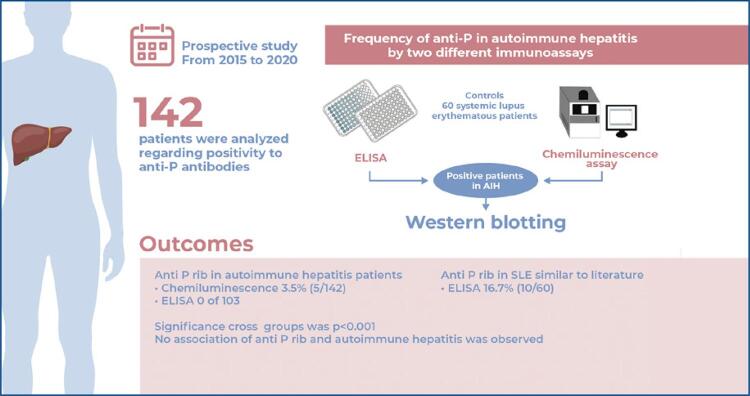
Visual Abstract

## INTRODUCTION

Specific and nonspecific autoantibodies are hallmarks of autoimmune liver diseases. ^( 1 )^ Autoimmune hepatitis (AIH) is an emblematic member of this disease group and is largely distributed worldwide, with a prevalence varying from 8.0-18.3/100,000, ^( 2 , 3 )^ with a ratio of 3.6 females/males affected. Despite wide distribution among races, European are predominantly affected. Clinically, a spectrum of presentations can be expected, ranging from acute hepatitis to chronic and overt liver disease, and one-fourth of the patients have advanced-stage liver fibrosis at the time of diagnosis. ^( 4 )^

The gold standard diagnostic methods remain the histopathological findings on liver biopsy, which are characterized by interface hepatitis, lymphoplasmacytic infiltrates, and rosette formation. ^( 5 )^ Autoantibodies are also important in diagnosis and include anti-smooth muscle antibodies (ASM; actin-F fraction), antinuclear antibodies (ANA), anti-liver and kidney microsomal antibody type-1 (LKM1), and anti-soluble liver antigen (SLA-LP) antibodies, sometimes in association with other autoantibodies at lower frequencies. ^( 1 )^

Regulatory T cell imbalance leads to increased production of Th17 cells, ^( 6 )^ cytotoxicity, apoptosis, necroptosis, and antibody production, causing perennial stimulus of the inflammatory cascade that results in hepatic tissue lesions, consequently evolving to fibrosis and cirrhosis. ^( 5 , 7 )^ Part of these mechanisms, as well as some clinical features, are remarkably similar in AIH and other systemic autoimmune diseases, particularly systemic lupus erythematous (SLE).

One of the important biomarkers in SLE associated with specific disease features is the anti-ribosomal P protein antibody (anti-P). This autoantibody is found in 6–46% of patients with SLE and is associated with specific manifestations of the disease, such as type V nephritis, hepatitis, and neuropsychiatric involvement. ^( 8 )^ Based on several similar features of these two autoimmune diseases, the possibility that anti-P could also be present in non-SLE associated AIH has been considered. Calich et al. ^( 9 )^ found anti-P in nine of 93 (9.7%) patients with non-SLE-associated AIH, and anti-P was associated with a higher frequency of cirrhosis in the long-term follow-up of these patients. However, these findings have not been confirmed in other studies. ^( 10 - 12 )^

## OBJECTIVE

To evaluate the frequency of anti-P antibodies in patients with autoimmune hepatitis.

## METHODS

### Study design

This prospective study evaluated the presence of anti-P in patients with AIH. The study was conducted at the Gastroenterology and Hepatology Division, *Universidade Federal de São Paulo* , Brazil and was approved by the Institutional Ethics Committee under CAAE: 49776615.0.0000.5505; # 1.524.672.

### Patients

Patients diagnosed with AIH based on the criteria of the International Autoimmune Hepatitis Group ^( 13 )^ were consecutively recruited between 2015 and 2020. Patients with overlapping syndromes and other associated causes of liver disease (HBV virus, HCV virus, alcohol, NASH) were excluded. A cohort of patients with a confirmed diagnosis of SLE, followed by the Rheumatology Division of the same university, was included as the Control Group.

Sex, age, AIH classification, autoantibody positivity, and stage of fibrosis at presentation were also recorded. The fibrosis stage was determined by the histological analysis of liver biopsies or by evident clinical signs of cirrhosis.

### Antibody measurements

The following autoantibody levels were determined: ANA, using standard indirect immunofluorescence on HEp-2 cells (HEp-2 IFA); anti-smooth muscle (SMA), anti-mitochondrial (AMA), and LKM-1, using indirect immunofluorescence (IIF) in rodent tissue. ^( 1 )^

Two different methods were used to evaluate the presence of anti-P antibodies, and their concordance was analyzed: chemiluminescence assay (CLIA) and enzyme-linked immunosorbent assay (ELISA). For CLIA, the QUANTA Flash ^®^ Ribosomal P kit (Inova Diagnostics, San Diego, CA, USA) was used according to the manufacturer’s instructions. Paramagnetic beads coated with antigenic determinants were incubated with the patient serum. After washing, an isoluminol-conjugated anti-human IgG antibody was added to the beads. After washing as before, the activating buffer was added, changing isoluminol to luminol, and the luminescence produced was measured as relative light units - chemiluminescent units (CU)-using a BIO-FLASH optical system (Biokit, Werfen Medical LTDA, Barcelona, Spain).

For ELISA, the QUANTA LiteTM Ribosome P Kit (Inova Diagnostics, San Diego, CA, USA) was used according to the manufacturer’s instructions. Patient serum was added to microplate wells coated with a P-dominant epitope for autoantibody binding. After washing, enzyme-conjugated anti-human IgG was added to the microplate. The enzyme substrate and chromogenic indicator were added to the wells and the color of the reaction was read by spectrophotometry.

Samples positive in any of the former immunoassays were processed by western blotting (WB), as previously reported. ^( 14 )^ Briefly, rabbit reticulocyte lysate was separated by 12% polyacrylamide gel electrophoresis and transferred to nitrocellulose sheets. Individual serum samples were diluted 1:100 with 5% slim milk (0.05%). Vertical nitrocellulose strips were incubated with tween 20 (25mL) and phosphate-buffered saline (PBS) for 30 minutes at room temperature. After washing with T-PBS, the strips were incubated for 30 minutes with peroxidase-conjugated goat anti-human IgG diluted 1:20,000 in MT-PBS. After washing, the strips were incubated with H _2_ O _2_ and a chromogenic solution. The reaction was interrupted with 1M H _2_ SO _4_ when optimal color development was achieved.

### Statistical analysis

Descriptive analysis was used to identify the frequency of positive results in the groups. The χ ^2^ test was used to compare AIH with SLE. An inference level of 5% (p<0.05) was considered significant. Analyses were conducted using the statistical software IBM SPSS (v23) and the free software Open Epi 3.0.

## RESULTS

During the study period, 177 AIH patients were enrolled. Thirty-five patients were excluded because of imprecise diagnosis, lack of data, or association with other causes of liver disease, resulting in a final cohort of 142 patients. The Control Group contained 60 patients with SLE. The general characteristics of the participants are presented in table 1 .

**Table 1:** Demographic, clinical, and laboratory characteristics of the 142 autoimmune hepatitis patients included in the study

Characteristic	
Age median and range (years)	47 (18–76)
Female gender, n (%)	129 (89.5)
Associated autoimmune diseases, n (%)	14 (10)
Type of AIH, n (%)	
Type 1, n (%)	135 (93.8)
Type 2, n (%)	9 (6.2)
Advanced liver fibrosis, n (%)	61 (42)
HEp-2 IFA positive, n (%)	119 (82.6)
SMA positive, n (%)	62 (43)
Anti-LKM-1 positive, n (%)	7 (5.2)
AMA positive, n (%)	11 (7.7)
IgG levels (mg/dL)	773-5307 (median 1696)

AIH: autoimmune hepatitis.

Chemiluminescence for anti-P was performed in all patients and was positive in five (3.5%). ELISA for anti-P was performed in 103 patients, including the five with positive CLIA, and was negative in all of them. The five anti-P-positive samples were tested in WB; one sample was negative (20.8 CU in CLIA), two were weakly positive (23.3 and 25.8 CU in CLIA), and two were positive (33.9 and 42.8 CU in CLIA). In the SLE Control Group, ten of 60 (17.7%) patients had anti-P antibodies detected by CLIA ( Table 2 ).

**Table 2:** Anti-P antibodies in patients with autoimmune hepatitis and systemic lupus erythematous

Anti-P assay	AIH			SLE		%	p value
	
	Positive	Negative	%	Positive	Negative		
CLIA	5*	137	3.5	10	50	16.7	<0.001
ELISA	0	103	0	-	-	-	

* Only three of these five patients had the anti-P antibody reactivity confirmed by western blot.

AIH: autoimmune hepatitis; SLE: systemic lupus erythematosus; CLIA: chemiluminescence assay; ELISA: enzyme-linked immunosorbent assay.

The number of patients with anti-P-positive AIH was too low to allow statistical comparison of clinical characteristics with anti-P-negative; however, some characteristics of these patients should be noted. Four of the five patients had histopathologically confirmed cirrhosis and one was scored at F2. Among patients with AIH, CU values ranged from 20.8 to 42.8 CU (median, 25 CU), and among SLE patients, from 31.7 to 60.3 (median, 33.5 CU).

## DISCUSSION

New markers for diagnosis and disease progression are extremely needed in patients with AIH to assist in establishing a correct diagnosis, determining disease progression, evaluating therapeutic interventions, defining treatment withdrawal, and estimating long-term survival. Furthermore, a better understanding of the triggers to the autoimmune process is required. ^( 7 , 8 )^

Some authors have investigated the presence of anti-P antibodies in autoimmune hepatic diseases based on their phenotypic and pathogenic similarities to SLE. The results to date have been conflicting, and thus this aspect requires further investigation.

The presence and impact of anti-P antibodies in patients with autoimmune liver diseases was analyzed using CLIA and ELISA anti-P immunoassays in 142 patients with AIH. The results were compared to those obtained in a Control Group of patients with SLE, where a 10-20% frequency of anti-P is expected, and up to 40% in severe disease. ^( 15 )^

The results showed that anti-P was uncommon and only observed in 5/142 (3.5%) patients with AIH, measured by CLIA. None of the patients had positive ELISA. The positive results observed in patients with AIH were confirmed by WB analysis in only 60% of them. For comparison, 60 patients with SLE were assayed using anti-P CLIA, and 10 (16.7%) had positive results. This frequency is within the expected range for SLE and demonstrates the appropriate performance of the CLIA in this study. The observed low frequency of anti-P antibodies in AIH confirms previous reports that this autoantibody specificity is possible in liver disease, albeit at a considerably lower frequency than that observed in SLE.

The pathogenesis of autoimmune diseases relies on three main pillars: genetic susceptibility, environmental triggers, and impairment of tolerance mechanisms, which ultimately lead to self-reactive T cell activation. Cytokines also play important roles in autoimmune processes and the consequent tissue damage. In SLE models, immune complex mediated inflammation is driven by pro-inflammatory cytokines, which are mainly produced in hepatocytes. Their presence was also verified in serum, strongly suggesting their involvement in the disease pathogenesis. Besides alterations to regulatory T cell populations, disruption in the immune system balance has also been demonstrated in AIH, driven by IL4, IL10, TGF beta, IL17, confirming similarities in pathogenic mechanisms of both diseases. ^( 6 , 16 , 17 )^

Thus, owing to the immunological and clinical similarities between AIH and SLE, the potential of anti-P as a biomarker for AIH has been considered. Although this hypothesis has been investigated previously, conflicting results have been reported. Some authors have described a positive association between more severe forms of AIH, whereas others have argued that methodological differences drive the contradictory results. ^( 9 , 10 - 12 )^ Considering that AIH is associated with immunogenetic patterns (DR3, DR4, DR7, and DR13) in different populations worldwide, we conducted a study in a cohort of Brazilian patients with AIH to confirm the previous results in our population. ^( 18 , 19 )^

There are studies comparing different techniques to detect anti-P antibodies, and despite the possibility of false-negative results with indirect immunofluorescence, high sensitivities have been reported for ELISA and immunoblotting, and these methods appear to have a reasonable correlation index. ^( 11 , 20 , 21 )^ These differences could be attributed, among other factors, to the fact that anti-P antibody detection is highly dependent on the antigenic epitopes used. Anti-P antibodies react with a conserved and common epitope at the carboxy-terminal domain of the three main ribosomal autoantigens, P0, P1, and P2. This short immunodominant epitope is widely used in solid-phase immunoassays such as ELISA, CLIA, and line blotting. It should be noted that some degree of disagreement among different methods is also observed in most of the autoantibody systems. As a general rule, positivity for a given autoantibody in more than one methodological platform confers higher clinical relevance to the result.

## CONCLUSION

Anti-P antibodies are detectable in autoimmune hepatitis, albeit at a considerably lower frequency than observed in systemic lupus erythematous. In addition, our results find an association with any particular presentation of the disease, underscoring the need for additional studies with a larger number of patients with heterogeneous clinical and pathological statuses.
